# A Conserved Function of *C. elegans* CASY-1 Calsyntenin in Associative Learning

**DOI:** 10.1371/journal.pone.0004880

**Published:** 2009-03-16

**Authors:** Frédéric J. Hoerndli, Michael Walser, Erika Fröhli Hoier, Dominique de Quervain, Andreas Papassotiropoulos, Alex Hajnal

**Affiliations:** 1 Institute of Zoology, University of Zürich, Zürich, Switzerland; 2 Division of Molecular Psychology, University of Basel, Basel, Switzerland; 3 Life Science Training Facility, Biozentrum, University of Basel, Basel, Switzerland; 4 Division of Psychiatry Research, University of Zürich, Zürich, Switzerland; 5 Center for Integrative Human Physiology, University of Zürich, Zürich, Switzerland; Columbia University, United States of America

## Abstract

**Background:**

Whole-genome association studies in humans have enabled the unbiased discovery of new genes associated with human memory performance. However, such studies do not allow for a functional or causal testing of newly identified candidate genes. Since polymorphisms in Calsyntenin 2 (CLSTN2) showed a significant association with episodic memory performance in humans, we tested the *C. elegans* CLSTN2 ortholog CASY-1 for possible functions in the associative behavior of *C. elegans*.

**Methodology/Principal Findings:**

Using three different associative learning paradigms and functional rescue experiments, we show that CASY-1 plays an important role during associative learning in *C. elegans*. Furthermore, neuronal expression of human CLSTN2 in *C. elegans* rescues the learning defects of *casy-1* mutants. Finally, genetic interaction studies and neuron-specific expression experiments suggest that CASY-1 may regulate AMPA-like GLR-1 glutamate receptor signaling.

**Conclusion/Significance:**

Our experiments demonstrate a remarkable conservation of the molecular function of *Calsyntenins* between nematodes and humans and point at a role of *C. elegans casy-1* in regulating a glutamate receptor signaling pathway.

## Introduction

The cellular and molecular mechanisms underlying learning and memory are the focus of intense research. Although many new components have been described that are conserved across different animal species, the exact mechanisms by which synaptic strength is regulated remain elusive [1] . Long-term potentiation (LTP) and depression (LTD), which are a key mechanisms underlying memory formation, involve plastic changes in synaptic strength through modulation of AMPA Glutamate receptor currents [2]. One frequently used mechanism by which neurons modulate synaptic strength is through the regulation of the number of neurotransmitter receptors at the surface of synapses [3]. Intracellular trafficking, exo- and endocytosis of receptors as well as surface dynamics also play important roles in regulating the exact number of receptors at the synapse [2], [4]. However, the exact mechanisms by which this is achieved are not completely understood.

Studies in both invertebrates and vertebrates have identified several genes and signaling pathways important for learning and memory. From this work it appears that many of the memory-related molecular mechanisms are conserved across different species. Despite the obvious differences in learning and memory tasks performed by different species and the anatomical differences between their nervous systems, recent human genetic studies suggest that genetic variability in the orthologs of related signaling molecules known from studies in model organisms contributes to inter-individual memory differences in humans [5]. Therefore, genes associated with human episodic memory identified in whole-genome association studies could provide new insights into the mechanisms underlying memory formation and storage.

Recently, an unbiased genome-wide screen for human hippocampus-dependent, episodic memory, which studied more than 500000 single nucleotide polymorphisms (SNPs), resulted in the identification of *CLSTN2* (encoding calsyntenin 2) as a memory-related human gene [6]. Specifically, *C* allele carriers of a common T→C substitution within *CLSTN2* had better episodic memory performance than *TT* genotype carriers in a verbal delayed recall task, which was performed by 341 Swiss young adults (median age 22 years). The better performance of the *C* allele carriers was observed 5 min and 24 h after learning, whereas immediate recall performance was similar between genotype groups, indicating that *CLSTN2* is related to hippocampus-dependent memory performance and that the findings were not biased by possible differences in motivation, attention and working memory performance between groups. This association was not replicated in a second population of middle-aged participants from the US, which may be partially attributed to differences in ethnicity, in mean age between study populations, or in differences between cognitive tasks used [6]. However, a recent independent study in adolescents replicated the beneficial effect of the *CLSTN2 C* allele on verbal recall [7].

Even though there exists no direct equivalent of human episodic memory in the small nematode *C. elegans*, several forms of associative behaviour and long-term memory have been observed in this model organism [8]–[11]. For example, *C. elegans* is capable of pairing food deprivation sensation with olfactory cues [12], gustatory cues [9] and the temperature of its environment [11] by using different sensory neurons and integrating interneurons. Essentially, this type of learning is akin to some classical conditioning paradigms such as conditioned taste aversion (CTA) where an unconditioned stimulus (US) is paired with a conditioned stimulus (CS) [13]. Moreover, *C. elegans* is capable of distinguishing multiple cues based on past experience using a serotonin dependent mechanism [8]. Together with an easily modifiable genetic background and many available knock-out alleles, *C. elegans* allows a fast and systematic way to analyze genes implicated in associative memory.

Taking advantage of the fact that the *C. elegans* genome encodes only one *CLSTN* gene (*casy-1*) homologous to vertebrate CLSTN2 and that a knock-out allele is available, we show that *casy-1* plays an important role in associative learning in both thermotaxis and chemotaxis conditioning paradigms. While this work was in progress, an independent study has identified *casy-1* in a forward genetic screen for behavioural mutants [14]. In addition to the reported behavioural defects of *casy-1* mutants, we show here that the pan-neuronal expression of human CLSTN2 rescues the chemotaxis conditioning defect of *casy-1(tm718)*, thus demonstrating a strong conservation between CLSTN2 and *casy-1* at the level of their molecular function. Finally, we describe a putative mechanism for CASY-1 in regulating associative behaviour via glutamate receptor signalling based on neuron-specific rescue experiments and on the genetic interaction between *casy-1* and the glutamate receptor subunit *glr-1*.

## Results and Discussion

### The *C. elegans* genome encodes a single CLSTN2 ortholog *casy-1*

**Figure 1:**
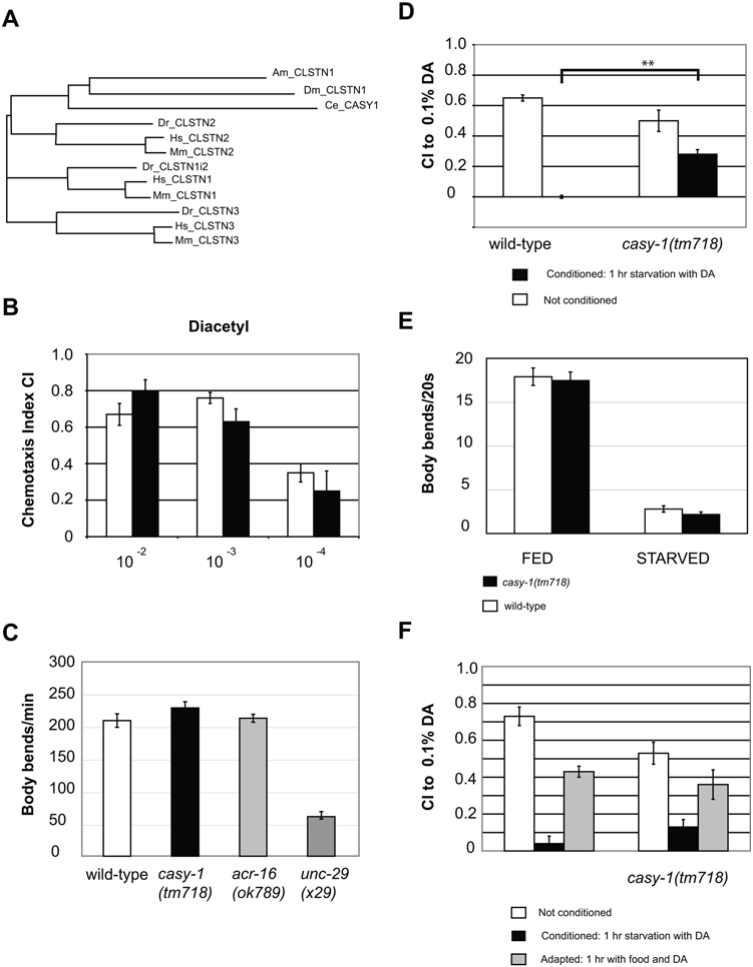
Olfactory associative learning defects in *casy-1(tm718)* mutants. (A) Rooted tree diagram showing the sequence similarities between the invertebrate and the three classes of vertebrate calsyntenins. The protein sequences of CLSTN1, CLSTN2 and CLSTN3 from *Homo sapiens* (*Hs*), *Mus musculus* (*Mm*), *Danio rerio* (*Dr*) and the single calsyntenins from *Drosophila melanogaster* (*Dm*), *Apis mellifera* (*Am*) and *Caenorhabditis elegans* (*Ce*) were aligned using the ClustalX program, and a rooted tree was drawn using PHYLIP. Note that the invertebrate calsyntenins and the vertebrate CLSTN2 proteins originate from a common branch. (B) Chemotaxis of wild-type and *casy-1(tm718)* worms towards 10^−2^, 10^−3^ and 10^−4^ fold dilution of Diacetyl in 100% EtOH(V/v) assay in the absence of conditioning. The assays were repeated on three different days using one plate for each condition and were quantified using the chemotaxis Index CI (CI = (worms in DA - worms at EtOH)/ total number of worms, see methods). Error bars indicate the standard error of mean. White bars: wild-type N2, Black bars: *casy-1(tm718)*. (C) Swimming assay of *casy-1(tm718)*, wild-type, nicotinic Acetylcholine-receptor *acr-16* knock out (*ok789*) and levamisole acetylcholine-receptor *unc-29* subunit knock-out (*x29*). Number of body bends per minute counted manually, and blinded to the respective genotypes (N = 20). (D) Chemotaxis of starvation conditioned wild-type and *casy-1(tm718)* animals. The experiment was repeated on three separate days with six replicates per assay. The results of a Student t-test are indicated as * = p<0.05 and ** = p<0.01. (E) Food sensing assay. Locomotion rate of wild-type and *casy-1(tm718)* worms in body bends/20 seconds of worms transferred from a food plate to another food plate (FED), or worms allowed to starve on an empty agar plate for 1 hr (STARVED). White bars: wild-type, Black bars: *casy-1(tm718)*. (F) Adaptation assay. Comparison of the chemotaxis Index CI of wild-type and *casy-1(tm718)* to 0.1% DA after starving for 1 hour without DA (White bars), with 100% DA (Black Bars) and on food for 2 hours with 100% DA (Grey bars). Assays were repeated on two different days using 3 replicates per condition. For the complete dataset of the behavioral assays, see Table S1.

To test a causal relationship between CLSTN2 function and learning and memory, we searched the genomes of invertebrate model organisms for CLSTN2 orthologs. While vertebrate genomes typically encode three *Calsyntenin* family members, the genomes of invertebrates like *Drosophila melanogaster* and *C. elegans* contain only a single *Calsyntenin* gene (Fig. 1A). Protein sequence alignment of the three vertebrate Calsyntenin family members with the invertebrate Calsyntenins indicates that the single *C. elegans* homolog CASY-1 as well as *Drosophila* Calsyntenin are most similar to vertebrate CLSTN2 (Fig. 1A).

**Figure 2:**
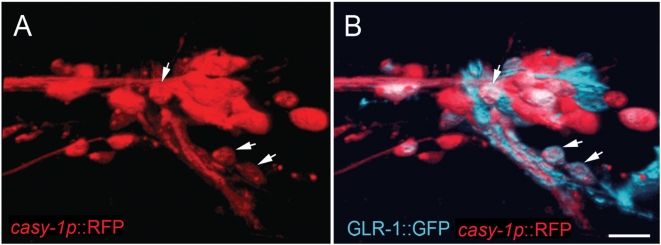
Expression pattern of a transcriptional *casy-1* reporter. (A) Expression of the *casy-1p::*RFP transcriptional reporter (red) and (B) a GLR-1::GFP translational reporter (blue) [22] in the nerve ring of an adult animal. A 3D reconstruction of confocal sections through the left hemisphere is shown (see methods). The two arrowheads in the bottom right corner point at RMDDL and SMDDL and the arrowhead in the top half points at SMDVL, which co-express *casy-1p::*RFP and GLR-1::GFP. Anterior is left and ventral is bottom. Scale bar in (B) is 10 µm.

CLSTN2 is a type I transmembrane protein with two extracellular calcium-binding cadherin domains and two intracellular kinesin light chain-binding domains [15], [16]. These domains are conserved in all three Calsyntenin family members including *C. elegans* CASY-1 [15]. Similar to mammalian Calsyntenins, a transcriptional *casy-1* reporter is expressed in many head nerve ring neurons, some of which send processes into the ventral nerve cord (Fig. 2A and data not shown). Moreover, a GFP-tagged CASY-1 protein was reported to localize at synapses (Duan and Hedgecock, personal communication). Given the sequence similarity between human CLSTN2 and *C. elegans* CASY-1 and their neuronal expression in both organisms, we asked whether the *casy-1* gene might function in regulating associative learning in *C. elegans*. The *casy-1* deletion mutant *tm718* (kindly provided by S. Mitani) contains a 601 bp deletion in exon 4, creating a frameshift followed by a premature stop codon. The *tm718* allele results in the production of a protein truncated at position 117 that lacks most of the extracellular and the entire intracellular domain. We observed no obvious anatomical, behavioral or locomotory defects in naive *casy-1(tm718)* animals (Fig. 1B,C). Moreover, *casy-1(tm718)* animals appear healthy and are fertile.

### Behavioral defects in *C. elegans casy-1* mutants

To test associative learning in *C. elegans*, we used three established context-dependent behavioral paradigms that are based on olfactory, gustatory and thermosensory starvation conditioning, respectively [11], [17]. The chemotaxis of naive *casy-1(tm718)* animals to three different volatile attractants was comparable to the response of the wild-type strain (Fig. 1B and Fig. S1). We thus investigated the olfactory associative learning capacity of *casy-1(tm718)* animals by testing their ability to reverse the attraction to an odorant after associating this odorant with a negative stimulus such as starvation (*see methods*). After starvation conditioning, the chemotaxis index (CI) of unconditioned controls and conditioned animals was compared in a quantitative chemotaxis assay [17]. Unconditioned wild-type and *casy-1(tm718)* animals both displayed strong chemotaxis to 0.1% diacetyl (DA, Fig. 1D), indicating that *casy-1(tm718)* mutants have no sensory defects in DA olfaction under these conditions. After a one hour starvation period in the presence of DA, wild-type animals did not show any attraction to DA, while *casy-1(tm718)* mutants were still significantly attracted by DA (CI = 0.3, p<0.01 using a Student t-test, 6 replicates repeated three times), albeit less efficiently than unconditioned control animals (Fig. 1D). The behavioral difference between wild-type and *casy-1(tm718)* animals is not due to a defect in food detection, since we observed normal slowing of *casy-1(tm718)* locomotion compared to wild-type, when animals were deprived of food and replaced on a new bacterial lawn (Fig.1E) [18].

To investigate the possibility that the chemotactic association defect of *casy-1(tm718)* could be due to adaptation (i.e. a decrement in response due to sensory fatigue that cannot be dishabituated [19]) rather than to an associative learning defect, we pre-exposed both strains to concentrated DA in the presence of abundant food before measuring their CI to 0.1% DA (gray bars in Fig. 1F). DA-adapted wild–type and *casy-1(tm718)* animals showed a similar partial reduction in their CI to DA , indicating that *casy-1(tm718)* mutants can adapt to high concentrations of DA. We thus conclude that a loss of *casy-1* function predominantly reduces associative learning without significantly impairing olfactory adaptation.

**Figure 3:**
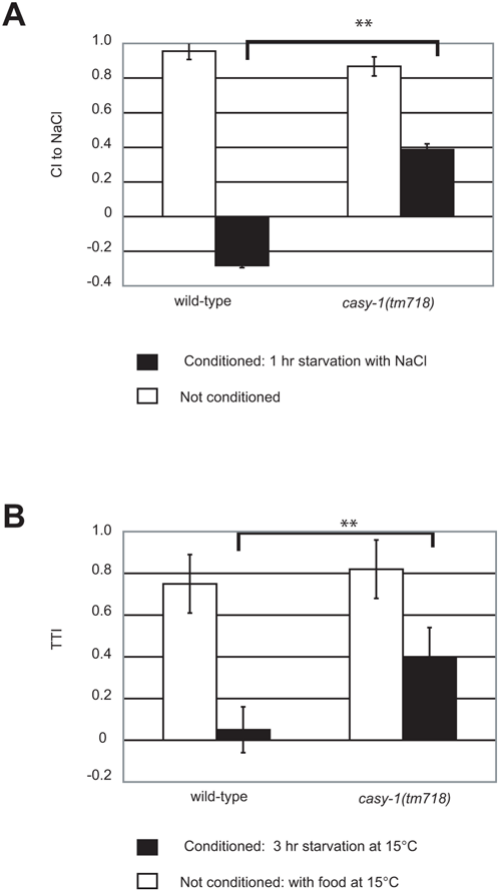
NaCl chemotaxis and thermotaxis associative learning defects in *casy-1(tm718)* mutants. (A) Chemotaxis of starvation conditioned wild-type (N2) and *casy-1(tm718)* worms to 25 mM NaCl. The chemotaxis index was calculated as CI = (worms at NaCl - worms at neutral)/ total number of worms. The experiment was repeated on three separate days with three replicates per assay. Error bars indicate the standard error of mean. (B) Thermotaxis association experiments with wild-type and *casy-1 (tm718)* animals. The thermotaxis index was calculated as TTI = (worms on the cold side of the plate – worms on the warm side)/ total worms in the assay. The experiment was repeated on three separate days. Error bars indicate the standard error of mean. In (A) and (B), the results of a Student t-test are indicated as * = p<0.05 and ** = p<0.01. For the complete dataset, see Table S1.

Next, we tested the performance of *casy-1(tm718)* mutants in an “gustatory” NaCl chemotaxis conditioning paradigm [9]. Wild-type worms display a strong attraction to 25 mM NaCl that is reversed when worms are first starved in the presence of NaCl in liquid cultures for 1 hour (Fig. 3A) [9]. Unconditioned *casy-1(tm718)* worms displayed a chemotaxis index (CI) that was similar to naive wild-type animals. However, when starved in the presence of NaCl *casy-1(tm718)* mutants did not show an aversion but only a partial decline in their attraction towards NaCl (Fig. 3A).

To test the associative behavior in the context of a third sensory system, we examined the performance of *casy-1(tm718)* mutants in a thermotaxis conditioning paradigm. Wild-type animals typically migrate towards the temperature at which they had been previously fed, but they avoid this temperature after a 3 hour starvation period [11]. We used a modified version of this conditioning paradigm by training groups of worms at specific temperatures and placing them on thin agar plates with a steep temperature gradient to measure their Thermotaxis Index (TTI) [20]. Wild-type worms grown at 15°C showed a TTI close to zero after 3 hours of starvation conditioning at 15°C, whereas *casy-1(tm718)* animals continued to exhibit significant albeit reduced thermotaxis to 15°C after starvation conditioning at this temperature (Fig. 3B).

In conclusion, *casy-1(tm718)* mutants exhibit strong associative learning defects in the context of three different sensory stimuli with no sensory impairment of the naive animals when compared to wild-type. These results point at a central function of CASY-1 in promoting associative learning downstream of different sensory stimuli.

### Expression of human CLSTN2 rescues the behavioral defects of *casy-1* mutants

**Figure 4:**
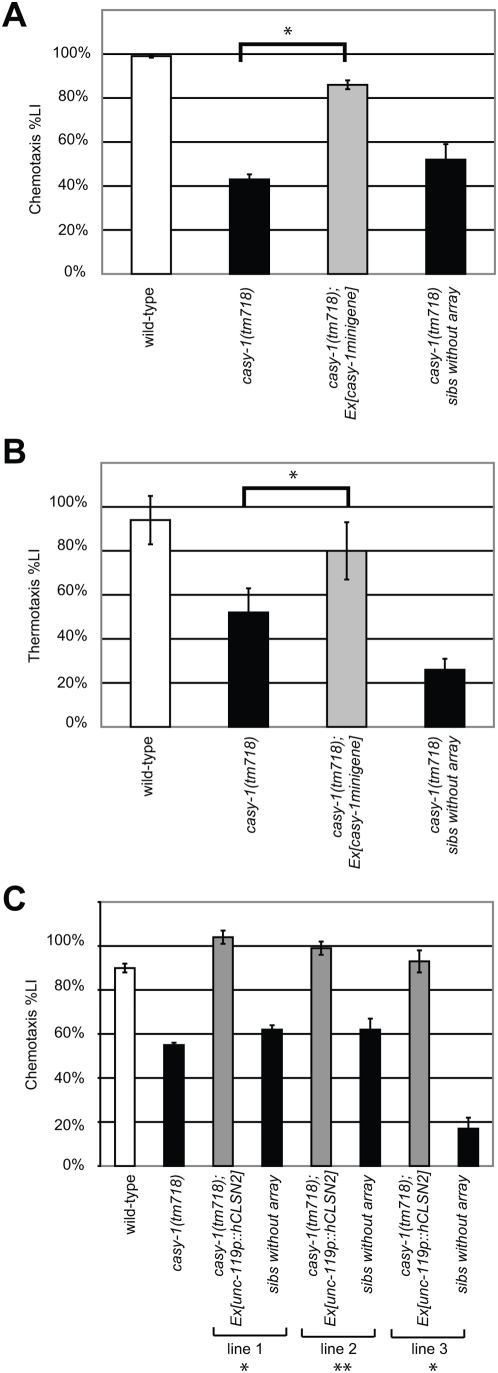
Rescue of the *casy-1(tm718)* behavioral defect with *C. elegans casy-1* and human *clstn2* transgenes. (A) Rescue of the chemotaxis and (B) thermotaxis conditioning defects with a *casy-1* minigene. Results obtained with one (*zhEx242.1*) of four transgenic lines are shown. To quantify the rescue, we defined a % Learning Index as %LI = 100 ^.^ (CI of naive worms - CI of conditioned worms)/ CI of naive worms) and analogous for the TTI. As controls, *casy-1(tm718)* animals that had lost the GFP-labeled extrachromosomal rescuing array (*casy-1(tm718)* sibs without array) were included with the transgenic animals in the assay, and their %LI was scored in parallel with the %LI of the transgenic animals. (C) Rescue of the *casy-1(tm718)* chemotaxis conditioning defects by expression of human CLSTN2 cDNA under control of the neuronal *unc-119* promoter and the *casy-1* 3′UTR. The results obtained with three independent lines *zhEx282.1* to *zhEx282.3* are shown. In (C), Student t-test LIs from *casy-1(tm718)* were compared to LIs of the rescue lines. For the complete dataset, see Table S1 and Fig. S2.

To confirm that the olfactory and thermotaxis association defects observed in *tm718* animals are due to the loss of *casy-1* function, we introduced a *casy-1* minigene composed of 5 kb of 5′ regulatory sequences fused to 3 kb cDNA of the long *casy-1* isoform (B0034.3a) and 3 kb of 3′ non-coding sequences into *casy-1(tm718)* animals. A transgenic line carrying the *casy-1* minigene on an extrachromosomal array (*zhE242.1[casy-1 minigene]*) was tested in the olfactory and thermotaxis conditioning paradigms. We calculated a learning index (%LI) as the difference between the CI or TTI of unconditioned and conditioned animals divided by the CI or TTI, respectively, of the unconditioned animals [8] (see methods). In both paradigms, the transgenic animals showed significant rescue of the %LI, while their non-transgenic siblings (*casy-1(tm718)* sibs without array) that were simultaneously scored on the same assay plates exhibited behavioral defects comparable to the parental *casy-1(tm718)* strain (Fig. 4A, B).

To test the functional conservation between human CLSTN2 and *C. elegans* CASY-1 at the molecular level, we expressed human CLSTN2 cDNA under the control of the pan-neuronal *unc-119* promoter and with the *C. elegans casy-1* 3′UTR in place of the CLSTN2 3′UTR in *casy-1(tm718)* mutants and measured the %LI of CLSTN2 transgenic animals using the olfactory conditioning assay. All three transgenic lines that were tested showed a significant rescue of the behavioral defects (Fig. 4C). Control transgenic animals carrying the *unc-119* promoter-*casy-1* 3′UTR vector lacking the CLSTN2 cDNA insert exhibited no significant increase in the %LI when compared to non-transgenic *casy-1(tm718)* animals (Fig. S2). Thus, human CLSTN2 can functionally replace *C. elegans* CASY-1 in an associative learning paradigm.

### CASY-1 acts in a GLR-1 Glutamate receptor pathway

**Figure 5:**
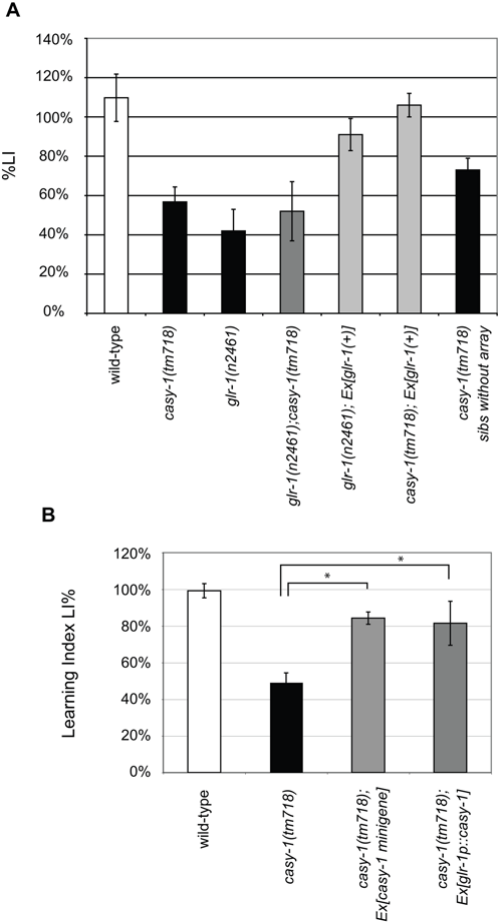
Genetic interaction between *casy-1* and the *glr-1* glutamate receptor signaling pathway. (A) Chemotaxis conditioning assays with *casy-1(tm718) and glr-1(n2461)* single and the double mutants and rescue of *casy-1(tm718)* conditioning defects by over-expression of *glr-1* using the *zhEx243.1* array. (B) Rescue of the *casy-1(tm718)* conditioning defects by expression of *casy-1* cDNA under control of the *glr-1* promoter. The average %LI of four independent lines is shown. Two of the lines showed a complete and two lines a partial rescue of the %LI. For comparison, the data for the *casy-1* minigene rescue experiment from fig. 4 A are shown. The scoring and quantifications were done as described in the legend to Fig. 4. For the complete dataset, see Table S1.

Human CLSTN1 and CLSTN2 form a complex with the MINT2/X-11-like neuronal adaptor protein and kinesin light chain (KLC1), suggesting a function for CLSTNs in the transport or sorting of synaptic vesicles [15], [16], [21]. Since mutations in the *C. elegans* ortholog of Mint2 (*lin-10*) cause defects in the clustering of the AMPA-type glutamate receptor subunit GLR-1 at the synapses of ventral cord interneurons and LIN-10 can bind to the PDZ binding motif at the C-terminus of GLR-1 [22], we hypothesized that CASY-1 might regulate the synaptic function or transport of GLR-1. Even though we did not observe a significant mislocalization of a translational GLR-1::GFP reporter in ventral cord motorneurons of *casy-1(tm718)* mutants (data not shown), *glr-1(n2461)* mutants showed similar association defects in the olfactory conditioning assays as *casy-1(tm718)* mutants (Fig. 5A). Notably, GLR-1 has been previously shown to be important for olfactory assocation and critical for long-term memory in *C. elegans* [23], [24]. To test a possible function of CASY-1 in a GLR-1 signaling pathway, we examined the genetic interaction between *casy-1(tm718)* and *glr-1(n2461)*. We found no further reduction in the %LI in the *casy-1(tm718); glr-1(n2461)* double loss-of-function mutant compared to either single mutant, suggesting that *casy-1* and *glr-1* may act in the same pathway regulating olfactory conditioning (Fig. 5A). We thus tested if increased levels of GLR-1 could rescue the behavioral defects of *casy-1(tm718)* mutants. For this purpose, we introduced a rescuing multicopy extrachromosomal array containing a 6 kb fragment spanning the *glr-1* locus (*zhEx243.1[glr-1(+)]*) into the *casy-1(tm718)* background. *casy-1(tm718); zhEx243.1[glr-1(+)]* animals showed a similar %LI in the olfactory conditioning assay as wild-type animals (Fig. 5A). Thus, increasing the GLR-1 gene dosage can compensate for the behavioral defects of *casy-1(tm718)* mutants, suggesting that CASY-1 positively regulates GLR-1 signaling during olfactory conditioning.

Some of the head neurons expressing the *casy-1* transcriptional reporter also expressed the *glr-1::gfp* reporter (Fig. 2B). Strongest co-expression was seen in the RMDD, SMDD, RMD and SMDV motor/interneurons that regulate head turning, and weaker *casy-1p::rfp* expression was observed in the *glr-1*-positive AVE command interneurons (not visible in Fig. 2B). We therefore tested if expression of *casy-1* under control of the *glr-1* promoter was sufficient to rescue the olfactory learning defects of *casy-1 (tm718)* mutants. In two out of four lines tested, the DA starvation conditioning defect was completely and in the remaining two lines weakly rescued (Fig. 5B and Table S1). Thus, *casy-1* acts at least in part in *glr-1* positive neurons during olfactory associative learning. It is interesting to note that Ikeda et al.[14] found that during salt chemotaxis conditioning, expression of *casy-1* in *glr-1* positive neurons was not sufficient to rescue the associative learning defects. Accordingly, a recent study by Kano et al. [25] showed that associative learning as well as short-term memory using the salt chemotaxis conditioning paradigm are not *glr-1* dependent. Thus, *casy-1* may perform another, *glr-1* independent function during gustatory (salt) chemotaxis learning, as *casy-1* may act in multiple, distinct pathways depending on the type of sensory inputs that need to be associated with the starvation signal.

### Conclusions

In summary, our study reveals an important role of *C. elegans casy-1 calsyntenin* in associative learning in response to different environmental stimuli. It should be noted that in all the association assays shown, the behavior of conditioned *casy-1(tm718)* mutants still significantly differed from the naive controls (i.e. the %LI of *casy-1* mutants was always greater than 0), indicating that loss of *casy-1* function does not result in a complete loss of all associative behavior. Thus, there must exist multiple parallel pathways controlling associative learning in *C. elegans*. For example, components of the insulin signaling pathway have been implicated in salt chemotaxis learning, and *casy-1* was found to act in parallel to the insulin pathway during salt chemotaxis learning [14].

Finally, we demonstrate that the molecular function of human CLSTN2 and *C. elegans* CASY-1 is conserved, as human Calsyntenin-2 can functionally replace CASY-1 during olfactory learning. Given the relatively large evolutionary distance between these two species and the anatomical dissimilarity of their nervous systems, this degree of conservation at the molecular level is remarkable. Thus, Calsyntenin might be a key component of conserved molecular pathways regulating different aspects of learning and memory in diverse species.

## Methods

Strains were maintained and grown according to standard procedures [26]. Wild-type refers to *C. elegans* Bristol, variety N2. *casy-1(tm718)* mutants were kindly provided by the Mitani Lab and backcrossed three times before use in all assays. All transgenic animals were generated by microinjection of the indicated DNAs into the syncytial gonads as described. Alleles and transgenes used: LGI: *unc-29(x29)* (kind gift of A.V. Maricq); LGII: *casy-1(tm718)*; LGIII: *glr-1(n2461)*; LGV: *acr-16(ok789)*; transgenes: *zhEx242.1[casy-1 minigene; sur-5::gfp]*, *zhEx243.1[glr-1(+), lin-48 ::gfp]*, *zhEx282.1 to 282.3[unc-119p::*CLSTN2*::casy-1 3′UTR, sur-5::gfp]*, *zhEx285.1 to zhEx285.3[unc-119p::no insert::casy-1 3′UTR, sur-5::gfp]*, *zhEx245[casy-1p::rfp]*, *nuIs24[glr-1::gfp]*, *Ex[glr-1p::casy-1]*.

### PCR fusion constructs

All DNA fragments were amplified using proof reading polymerase from *C. elegans* genomic DNA or total N2 cDNA. Individual fragments were fused by PCR fusion [27]. A 6 kb genomic *glr-1* fragment was amplified (forward: 5′-ccggtcatacgggagataga-3′, reverse: 5′- taaattttcctgggggcttc-3′) to generate *zhEx243.1*. 5 kb of the 5′ UTR region of *casy-1* (forward outer: 5′- ggatattggtcaccttcccta-3′, nested forward: 5′- ttctagattattctgacaaccatttg-3′, reverse: 5′-cgagcagcatggtgatgtttg-3′) were fused to 2995 bp *casy-1* cDNA (B0034.3a, 5′ fusion primer: 5′-actcacgcacacaaaaccaatcatgcgaactgcgtactttatttttgtc-3′, reverse: 5′- ggagggagtcatgaatgttga-3′) and 1.6 kb of 3′UTR (forward 3′UTR: 5′-gttcgtttgacaagccgttt-3′, nested forward 3′UTR: 5′- agccgtttggtttttcaatg-3′, cDNA fusion primer: 5′- aattccttcaggcatgttgc-3′). This PCR construct was used together with the transformation marker *sur-5::gfp* to generate *zhEx242.1*. Details on the construction of the *glr-1p::casy-1*, the *casy-1p:.rfp* and the *unc-119p::*CLSTN2*::casy-1 3′UTR* rescue and control (without insert) constructs are available upon request.

### Olfactory conditioning

All assays were conducted with 50–200 well-fed synchronized young adult worms, using 10 cm Petri CTX agar dishes (2% agar, 5 mM KPO_4_ pH = 6.0, 1 mM CaCl_2_, 1 mM MgSO_4_). Except for agar composition, chemotaxis assays were performed as described previously [17]. Adaptation and starvation conditioning assays were performed as previously described [12], except that animals were washed three times with M9 buffer (22 mM KH_2_PO_4_, 22 mM Na_2_HPO_4_, 85 mM NaCl, 1 mM MgSO_4_) for 20 min each, resulting in 1 hour pre-starvation before the olfactory conditioning was performed.

### NaCl conditioning

Salt chemotaxis and salt chemotaxis learning assays were assessed as described before with some modifications [28], [29],. Briefly, synchronized and well-fed young adult nematodes were washed 3 times in CTX buffer. 100–200 worms were placed at the intersection of a four-quadrant CTX plate to test chemotaxis and liquid was removed with a tissue paper. Chemotaxis plates were prepared one day in advance. Pairs of opposite quadrants of four-quadrant Petri plates (Falcon X plate, Becton Dickinson Labware) were filled with 16 ml buffered agar (2% agar, 5 mM KPO_4_ pH 6, 1 mM CaCl_2_ and 1 mM MgSO_4_), either containing 25 mM NaCl or not. Adjacent quadrants were connected with a thin layer of molten agar 1 h before the assay. The chemotaxis index was calculated 10 min after the worms were placed on the CTX plates: (A–C)/ total number of worms), where A is the number of worms at the quadrants with, and C is the number of worms at the quadrants without NaCl.

For NaCl chemotaxis learning assays, the collected nematodes were transferred after the washing procedure into 30 ml CTX buffer containing 20 mM NaCl for 1 h at room temperature [14], and chemotaxis was tested immediately afterwards. All experiments were performed in triplicates at least three times.

### Thermotaxis conditioning

We created a thermotaxis setup as described previously using a steep thermal gradient on a thin agar plate [20]. A 2–3 mm thick CTX agar plate 130 mm long 90 mm wide was rested on heated and cooled metal blocks, respectively, such that 13°C was measured at one end and 33° at the other end of the plate. 200–400 worms were spotted along the 22°C isothermic line measured shortly before applying the worms. The worms were then left to migrate for 45 min. At the end of the assay, the plate was separated into a cold region and a warm region along the 22°C isothermic line, and the worms were immediately counted to determine the TTI as described [20].

### Microscopy

For the image shown in Fig. 2, animals were anesthetized with 10 mM NaN_3_ and mounted in M9 buffer on 3% agarose pads. Optical sections through the left hemisphere were recorded on a Leica SP2 confocal microscope using a 63× N.A. 1.4 objective and a z-step size of 0.73 µm. 3D reconstructions were generated using the volocity 2.3. software package (Improvision) and a lateral view is shown.

## Supporting Information

Figure S1 Naive chemotaxis of wild-type and casy-1(tm718) mutants. Chemotaxis of naive animals to volatile attractants (Diacetyl and Isoamyl alcohol) and a repellent (2-Nonanone) was quantified as described in the methods and the legend to Fig. 1. The error bars show the SEM.
(0.39 MB EPS)

Figure S2 Chemotaxis conditioning of casy-1(tm718) negative control lines. Chemotaxis conditioning transgenic of casy-1(tm718) carrying the unc-119 promoter-casy-1 3′UTR vector without cDNA insert (zhEx285.1 to zhEx285.3[unc-119p::no insert]). The average %LI of three independent control lines and their siblings without array is shown. The LI was calculated as described in the methods and the legend to Fig. 3 and is expressed as % value.
(0.38 MB EPS)

Table S1 Supporting document
(0.52 MB PDF)
