# Supplementary material for: A Conserved Function of C. elegans CASY-1 Calsyntenin in Associative Learning
Source: PLoS One. 2009 Mar 16;4(3):e4880. doi: 10.1371/journal.pone.0004880 (PMC2654071; doi:10.1371/journal.pone.0004880)
Supplement: Table S1 — Supporting document (0.52 MB PDF) [file pone.0004880.s003.pdf]

Supplementary tables

Fig.1B

		diacetyl 10-2			diacetyl 10-3			diacetyl 10-4		
**wild-type**	Plates	**1**	**2**	**3**	**1**	**2**	**3**	**1**	**2**	**3**
	DA	105	103	73	119	214	172	43	40	41
	EtOH	0	0	0	1	0	3	14	5	10
	N	44	44	48	42	66	37	42	54	28
	total	149	147	121	162	280	212	99	99	79
	CI	0.70	0.70	0.60	0.73	0.76	0.80	0.29	0.35	0.39
	**Average**	**0.67**			**0.76**			**0.35**		
	**Standard deviation**	**0.06**			**0.03**			**0.05**		

		diacetyl 10-2			diacetyl 10-3			diacetyl 10-4		
casy-1(tm718)	Plates	**1**	**2**	**3**	**1**	**2**	**3**	**1**	**2**	**3**
	DA	19	31	30	14	20		20	20	15
	EtOH	0	0	0	1	2		7	10	3
	N	5	5	10	4	9		18	47	18
	total	24	36	40	19	31		45	77	36
	CI	0.79	0.86	0.75	0.68	0.58		0.29	0.13	0.33
	**Average**	**0.80**			**0.63**			**0.25**		
	**Standard deviation**	**0.06**			**0.07**			**0.11**		

Fig. 1D

wild-type

Conditions	Unconditioned																			
Trials	day1						day2						day3							
Plates	1	2	3	4	5	6	1	2	3	4	5	6	1	2	3	4	5	6		
DA	88	72	82	61	100	65	107	72	114	71	37	59	76	78	83	64	66	80		
EtOH	9	16	16	8	9	8	5	6	9	1	2	1	1	4	0	2	4	3		
N	1	36	26	25	23	35	17	10	20	28	22	18	24	31	44	44	45	44		
total	98	124	124	94	132	108	129	88	143	100	61	78	101	113	127	110	115	127		
CI	0.81	0.45	0.53	0.56	0.69	0.53	0.79	0.75	0.73	0.70	0.57	0.74	0.74	0.65	0.65	0.56	0.54	0.61		
**Average**	**0.65**																			
**SEM**	**0.02**																			
Conditions	DA Conditioned																			
Trials	day1						day2						day3							
Plates	1	2	3	4	5	6	1	2	3	4	5	6	1	2	3	4	5	6		
DA	2	8	10	5	19	14	13	8	18	4	5	11	10	2	3	6	4	9		
EtOH	2	7	7	4	19	16	18	12	13	4	7	10	4	3	2	6	7	3		
N	50	75	119	66	90	126	110	97	91	31	65	101	85	60	73	88	81	75		
total	54	90	136	75	128	156	141	117	122	39	77	122	99	65	78	100	92	87		
CI	0.00	0.01	0.02	0.01	0.00	-0.01	-0.04	-0.03	0.04	0.00	-0.03	0.01	0.06	-0.02	0.01	0.00	-0.03	0.07		
**Average**	**0.00**																		**% LI**	**99**
**SEM**	**0.01**																		**SEM**	

casy-1(tm718)

Conditions	Unconditioned																			
Trials	day1						day2						day3							
Plates	1	2	3	4	5	6	1	2	3	4	5	6	1	2	3	4	5	6		
DA	56	152	51	105	191	95	110	76	40	49	44	80	63	62						
EtOH	49	45	23	52	62	35	28	20	35	2	2	5	3	0						
N	29	29	16	27	46	28	26	22	36	8	5	11	11	10						
total	134	226	90	184	299	158	164	118	111	59	51	96	77	72						
CI	0.05	0.47	0.31	0.29	0.43	0.38	0.50	0.47	0.05	0.80	0.82	0.78	0.78	0.86						
**Average**	**0.50**																			
**SEM**	**0.07**																			
Conditions	DA conditioned																			
Trials	day1						day2						day3							
Plates	1	2	3	4	5	6	7	8	9	1	2	3	4	5						
DA	16	23	26	43	34	40	34	34	31	30	44	49	39	37						
EtOH	10	3	16	15	5	9	13	21	12	8	10	12	11	18						
N	25	43	45	36	40	43	34	32	34	20	30	32	26	30						
total	51	69	87	94	79	92	81	87	77	58	84	93	76	85						
CI	0.12	0.29	0.11	0.30	0.37	0.34	0.26	0.15	0.25	0.38	0.40	0.40	0.37	0.22						
**Average**	**0.28**																		**% LI**	**43**
**SEM**	**0.03**																		**SEM**	

Fig.1F

wild-type

Conditions	unconditioned					
Trials	day1			day2		
Plates	1	2	3	1	2	3
DA	31	9	10	113	125	37
EtOH	0	0	0	7	7	1
N	2	1	2	28	58	7
total	34	12	15	149	192	48
CI	0.91	0.75	0.67	0.71	0.61	0.75
**Average**	**0.73**					
**SEM**	**0.05**					
Conditions	DA starving conditioned					
Trials				day2		
Plates				1	2	3
DA				12	5	15
EtOH				4	4	14
N				69	115	126
total				86	126	158
CI				0.09	0.01	0.01
**Average**				**0.04**		
**SEM**				**0.04**		
Conditions	DA conditioned on food					
Trials	day1			day2		
Plates	1	2	3	1	2	3
DA	38	92	62	101	67	79
EtOH	14	27	13	27	16	14
N	18	31	24	77	23	31
total	71	152	102	206	108	127
CI	0.34	0.43	0.48	0.36	0.47	0.51
**Average**	**0.43**					
**SEM**	**0.03**					

casy-1(tm718)

Conditions	unconditioned					
Trials	day1			day2		
Plates	1	2	3	1	2	3
DA	43	57	35	46	101	141
EtOH	2	10	1	6	11	26
N	14	28	9	46	84	94
total	60	97	48	99	198	264
CI	0.68	0.48	0.71	0.40	0.45	0.44
**Average**	**0.53**					
**SEM**	**0.06**					
Conditions	DA starving conditioned					
Trials				day2		
Plates				1	2	3
DA				22	31	36
EtOH				4	11	18
N				69	136	142
total				96	180	199
CI				0.19	0.11	0.09
**Average**				**0.13**		
**SEM**				**0.04**		
Conditions	DA conditioned on food					
Trials	day1			day2		
Plates	1	2	3	1	2	3
DA	105	82	53	18	81	47
EtOH	21	4	19	0	0	3
N	55	26	54	71	109	138
total	182	114	129	90	192	191
CI	0.46	0.68	0.26	0.20	0.42	0.23
**Average**	**0.38**					
**SEM**	**0.08**					

Fig. 3A

wild-type

Conditions	Unconditioned								
Trials	day1			day2			day3		
Plates	1	2	3	1	2	3	1	2	3
NaCl	161	130	29	138	280	186	303	316	263
control	7	1	0	7	5	0	6	16	5
total	168	131	29	145	285	186	309	332	268
CI	0.92	0.98	1.00	0.90	0.96	1.00	0.96	0.90	0.96
**Average**	**0.96**								
**SEM**	**0.02**								

Conditions	Conditioned								
Trials	day1			day2			day3		
Plates	1	2	3	1	2	3	1	2	3
NaCl	108	182	62	98	118	146	117	126	71
control	126	309	195	229	271	206	159	189	153
total	234	491	257	327	389	352	276	315	224
CI	-0.08	-0.26	-0.52	-0.40	-0.39	-0.17	-0.15	-0.20	-0.37
**Average**	**-0.28**								
**SEM**	**0.08**								

casy-1(tm748)

Conditions	Unconditioned								
Trials	day1			day2			day3		
Plates	1	2	3	1	2	3	1	2	3
NaCl	49	47	33	169	101	106	170	178	139
control	11	5	2	6	5	9	2	9	6
total	60	52	35	175	106	115	172	187	145
CI	0.63	0.81	0.89	0.93	0.91	0.84	0.98	0.90	0.92
**Average**	**0.87**								
**SEM**	**0.06**								

Conditions	Conditioned								
Trials	day1			day2			day3		
Plates	1	2	3	1	2	3	1	2	3
NaCl	193	83	245	372	71	55	89	96	90
control	71	26	139	148	14	28	36	77	53
total	264	109	384	520	85	83	125	173	143
CI	0.46	0.52	0.28	0.43	0.67	0.33	0.42	0.11	0.26
**Average**	**0.39**								
**SEM**	**0.10**								

Fig. 3 B

	Conditions	Unconditioned			Conditioned		
**wild-type**	Trials	day1	day2	day3	day1	day2	day3
	Cold	66	170	182	68	n.d.	135
	Warm	3	12	65	82		92
	total	69	182	247	150		227
	TTXi	0.91	0.87	0.47	-0.09		0.19
	**Average**	**0.75**			**0.05**		
	**SEM**	**0.14**			**0.14**		

	Conditions	Unconditioned				Conditioned			
casy-1(tm718)	Trials	day1	day2	day3	day4	day1	day2	day3	day4
	Cold	169	52	89	81	40	33	21	114
	Warm	50	0	13	0	24	20	12	11
	total	219	52	102	81	64	53	33	125
	TTXi	0.54	1.00	0.75	1.00	0.25	0.25	0.27	0.82
	**Average**	**0.82**				**0.40**			
	**SEM**	**0.11**				**0.14**			

Fig. 4A

wild-type **% LI= 99± (from Fig. 3B)**

casy-1(tm718) **% LI= 43± (from Fig. 3B)**

Strain												
casy-1(tm718); zhEx242.1[casy-1minigene]	Conditions	Unconditioned										
	Trials	day 1			day 2			day 3				
	Plates	1	2	3	1	2		1	2	3		
	DA	10	6	4	10	25	5	19	35	35		
	EtOH	1	1	0	1	0	0	0	1	5		
	N	0	3	1	0	7	3	4	7	14		
	total	11	10	5	11	32		23	43	54		
	CI	0.82	0.50	0.80	0.82	0.78		0.83	0.79	0.56		
	**Average**	**0.74**										
	**SEM**	**0.05**										
	Conditions	Conditioned										
	Trials	day 1			day 2			day 3				
	Plates	1	2	3	1	2		1	2	3		
	DA	21	43	9	3	9	43	5	15	15		
	EtOH	14	26	7	3	6	26	2	9	2		
	N	52	81	19	11	33	81	19	78	22		
	total	87	150	35	17	48	150	26	102	39		
	CI	0.08	0.11	0.06	0.00	0.06	0.11	0.12	0.06	0.33		
	**Average**	**0.10**									**% LI**	**86**
	**SEM**	**0.03**										

Strain												
casy-1(tm718) sibs without array	Conditions	Unconditioned										
	Trials	day 1			day 2			day 3				
	Plates	1	2	3	1	2		1	2	3		
	DA	20	20	33	8	2						
	EtOH	1	1	4	0	0						
	N	6	6	30	4	10						
	total	27	27	67	12	12						
	CI	0.70	0.70	0.43	0.67	0.17						
	**Average**	**0.53**										
	**SEM**	**0.10**										
	Conditions	Conditioned										
	Trials	day 1			day 2			day 3				
	Plates	1	2	3	1	2		1	2	3		
	DA	19	19	16	18	5						
	EtOH	1	1	2	11	2						
	N	50	50	12	52	7						
	total	70	70	30	81	14						
	CI	0.26	0.26	0.47	0.09	0.21						
	**Average**	**0.26**									**% LI**	**52**
	**SEM**	**0.06**										

Fig. 4B

wild-type **% LI= 94 (from Fig. 3C)**
casy-1(tm718) **% LI= 52 (from Fig. 3C)**

casy-1(tm718); zhEx242.1[casy-1minigene]

Conditions	Unconditioned		
Trials	day1	day2	day3
Cold	17	15	14
Warm	5	0	0
total	22	15	14
TTI	0.55	1.00	1.00
**average**	**0.85**		
**SEM**	**0.15**		

Conditions	conditioned				
Trials	day1	day2	day3		
Cold	4	7	8		
Warm	4	5	4		
total	8	12	12		
TTI	0.00	0.17	0.33		
**average**	**0.17**			**% LI**	**80**
**SEM**	**0.10**				

casy-1(tm718) sibs without array

Conditions	Unconditioned		
Trials	day1	day2	day3
Cold	81	133	169
Warm	0	0	0
total	81	133	169
TTI	1.00	1.00	1.00
**average**	**1.00**		
**SEM**	**0.00**		

Conditions	conditioned				
Trials	day1	day2	day3		
Cold	114	132	109		
Warm	11	34	11		
total	125	166	120		
TTI	0.82	0.59	0.82		
**average**	**0.74**			**% LI**	**26**
**SEM**	**0.08**				

Fig. 4C

wild-type	Conditions	Unconditioned										
	trials	day 1			day 2			day 3				
	Plates	1	2	3	1	2		1	2	3		
	DA	46	43	92	67	26		68	130	342		
	EtOH	0	0	15	4	2		6	8	41		
	N	5	5	32	55	29		30	30	101		
	total	51	48	139	126	57		104	168	484		
	CI	0.90	0.90	0.55	0.50	0.42		0.60	0.73	0.62		
	**Average**	**0.65**										
	**SEM**	**0.06**										
	Conditions	Conditioned										
	trials	day 1			day 2			day 3				
	Plates	1	2	3	1	2		1	2	3		
	DA	16	21	23	47	9		85	86	37		
	EtOH	9	18	11	31	6		54	61	32		
	N	56	88	67	111	43		249	211	114		
	total	81	127	101	189	58		388	358	183		
	CI	0.09	0.02	0.12	0.08	0.05		0.08	0.07	0.03		
	**Average**	**0.07**									**% LI**	**90**
	**SEM**	**0.01**										

casy-1(tm718)	Conditions	Unconditioned										
	trials	day 1			day 2			day 3				
	Plates	1	2	3	1	2		1	2	3		
	DA	107	30	97	23	25		107	30	97		
	EtOH	21	6	27	2	0		21	6	27		
	N	95	18	69	63	72		95	18	69		
	total	223	54	193	88	97		223	54	193		
	CI	0.39	0.44	0.36	0.24	0.26		0.39	0.44	0.36		
	**Average**	**0.36**										
	**SEM**	**0.03**										
	Conditions	Conditioned										
	trials	day 1			day 2			day 3				
	Plates	1	2	3	1	2		1	2	3		
	DA	60	25	16	13	4		60	25	16		
	EtOH	31	12	0	0	0		31	12	0		
	N	120	47	59	54	39		120	47	59		
	total	211	84	75	67	43		211	84	75		
	CI	0.14	0.15	0.21	0.19	0.09		0.14	0.15	0.21		
	**Average**	**0.16**									**% LI**	**55**
	**SEM**	**0.01**										

casy-1(tm718); zhEx282.1[unc-119p::CLSTN2]	Conditions	Unconditioned										
		day 1			day 2			day 3				
	Plates	1	2	3	1	2	3	1	2	3		
	DA	7	2	10	5	5	2	2	9	6		
	EtOH	1	1	1	0	0	0	0	2	2		
	N	7	6	10	2	2	2	2	2	2		
	total	15	9	21	7	7	4	4	13	10		
	CI	0.40	0.11	0.43	0.71	0.71	0.50	0.50	0.54	0.40		
	**Average**	**0.48**										
	**SEM**	**0.06**										
	Conditions	Conditioned										
	trials	day 1			day 2			day 3				
	Plates	1	2	3	1	2	3	1	2	3		
	DA	0	0	2	0	0	0	6	10	10		
	EtOH	1	1	1	0	0	0	9	8	5		
	N	4	3	6	10	3	7	35	13	17		
	total	5	4	9	10	3	7	50	31	32		
	CI	-0.20	-0.25	0.11	0.00	0.00	0.00	-0.06	0.06	0.16		
	**Average**	**-0.02**									**% LI**	**104**
	**SEM**	**0.05**										

casy-1(tm718) sibs without *zhEx282.1* array	Conditions	Unconditioned										
		day 1			day 2			day 3				
	Plates	1	2	3	1	2	3	1	2	3		
	DA		18	34				8	16	21		
	EtOH		4	9				3	5	6		
	N		33	38				23	28	33		
	total		55	81				34	49	60		
	CI		0.25	0.31				0.15	0.22	0.25		
	**Average**	**0.24**										
	**SEM**	**0.02**										
	Conditions	Conditioned										
	trials	day 1			day 2			day 3				
	Plates	1	2	3	1	2	3	1	2	3		
	DA	3	3	10				20	10	9		
	EtOH	1	1	7				12	10	12		
	N	6	7	7				102	77	74		
	total	10	11	24				134	97	95		
	CI	0.20	0.18	0.13				0.06	0.00	-0.03		
	**Average**	**0.09**									**% LI**	**62**
	**SEM**	**0.03**										

casy-1(tm718); zhEx282.2[unc-119p::CLSTN2]	Conditions	Unconditioned										
	trials	day 1			day 2			day 3				
	Plates	1	2	3	1	2	3	1	2	3		
	DA	7	24	25	5	14	14	19	30	15		
	EtOH	6	6	23	1	4	5	2	9	1		
	N	8	9	9	3	3	3	4	10	3		
	total	21	39	57	9	21	22	25	49	19		
	CI	0.05	0.46	0.04	0.44	0.48	0.41	0.68	0.43	0.74		
	**Average**	**0.41**										
	**SEM**	**0.08**										
	Conditions	Conditioned										
	trials	day 1			day 2			day 3				
	Plates	1	2	3	1	2	3	1	2	3		
	DA	18	8	9	8	5		3	3	3		
	EtOH	17	11	5	6	5		3	1	10		
	N	15	8	9	13	4		39	40	32		
	total	50	27	23	27	14		45	44	45		
	CI	0.02	-0.11	0.17	0.07	0.00		0.00	0.05	-0.16		
	**Average**	**0.01**									**% LI**	**99**
	**SEM**	**0.04**										

casy-1(tm718) sibs without zhEx282.2 array	Conditions	Unconditioned										
	trials	day 1			day 2			day 3				
	Plates	1	2	3	1	2	3	1	2	3		
	DA	6	27	19	5	34	44	5	195	72		
	EtOH	1	6	0	27	3	7	27	29	6		
	N	48	44	68	17	39	37	73	185	34		
	total	55	77	87	49	76	88	105	409	112		
	CI	0.09	0.27	0.22	-0.45	0.41	0.42	-0.21	0.41	0.59		
	**Average**	**0.19**										
	**SEM**	**0.12**										
	Conditions	Conditioned										
	trials	day 1			day 2			day 3				
	Plates	1	2	3	1	2	3	1	2	3		
	DA	22	6	5	34	18		24	29	16		
	EtOH	12	8	13	8	6		11	11	7		
	N	69	38	28	57	30		141	143	186		
	total	103	52	46	99	54		176	183	209		
	CI	0.10	-0.04	-0.17	0.26	0.22		0.07	0.10	0.04		
	**Average**	**0.07**									**% LI**	**62**
	**SEM**	**0.05**										

casy-1(tm718); zhEx282.3[unc-119p::CLSTN2]	Conditions	Unconditioned										
	Trials	day 1			day 2			day 3				
	Plates	1	2	3	1	2	3	1	2	3		
	DA	53	16	8	10	9	27	4	4	5		
	EtOH	4	7	4	1	5	3	3	5	1		
	N	37	18	3	1	0	0	3	4	3		
	total	94	41	15	12	14	30	10	13	9		
	CI	0.52	0.22	0.27	0.75	0.29	0.80	0.10	-0.08	0.44		
	**Average**	**0.37**										
	**SEM**	**0.10**										
	Conditions	Conditioned										
	Trials	day 1			day 2			day 3				
	Plates	1	2	3	1	2	3	1	2	3		
	DA	13	12		13	6	13	3	3	1		
	EtOH	15	7		10	4	4	9	4	1		
	N	20	8		5	0	15	3	1	1		
	total	48	27	0	28	10	32	15	8	3		
	CI	-0.04	0.19		0.11	0.20	0.28	-0.40	-0.13	0.00		
	**Average**	**0.03**									**% LI**	**93**
	**SEM**	**0.08**										

casy-1(tm718) sibs without zhEx282.3 array	Conditions	Unconditioned										
	Trials	day 1			day 2			day 3				
	Plates	1	2	3	1	2	3	1	2	3		
	DA	16	6	6	5	38	63	46	33	29		
	EtOH	3	0	0	27	16	14	9	2	7		
	N	40	15	8	24	10	13	48	9	15		
	total	59	21	14	56	64	90	103	44	51		
	CI	0.22	0.29	0.43	-0.39	0.34	0.54	0.36	0.70	0.43		
	**Average**	**0.33**										
	**SEM**	**0.11**										
	Conditions	Conditioned										
	Trials	day 1			day 2			day 3				
	Plates	1	2	3	1	2	3	1	2	3		
	DA	2	1		49	19	52	7	20	71		
	EtOH	0	0		18	13	18	5	3	36		
	N	29	7		30	7	39	14	38	102		
	total	31	8		97	39	109	16	61	209		
	CI	0.06	0.13		0.32	0.15	0.31	0.75	0.28	0.17		
	**Average**	**0.27**									**% LI**	**17**
	**SEM**	**0.08**										

wild-type	Conditions	Unconditioned										
	Trials	day 1			day 2			day 3				
	Plates	1	2	3	1	2		1	2	3		
	DA	46	43	92	67	26		68	130	342		
	EtOH	0	0	15	4	2		6	8	41		
	N	5	5	32	55	29		30	30	101		
	total	51	48	139	126	57		104	168	484		
	CI	0.90	0.90	0.55	0.50	0.42		0.60	0.73	0.62		
	**Average**	**0.65**										
	**SEM**	**0.06**										
	Conditions	Conditioned										
	Trials	day 1			day 2			day 3				
	Plates	1	2	3	1	2		1	2	3		
	DA	16	21	23	47	9		85	86	37		
	EtOH	9	18	11	31	6		54	61	32		
	N	56	88	67	111	43		249	211	114		
	total	81	127	101	189	58		388	358	183		
	CI	0.09	0.02	0.12	0.08	0.05		0.08	0.07	0.03		
	**Average**	**0.07**									**% LI**	**90**
	**SEM**	**0.01**										

casy-1(tm718)	Conditions	Unconditioned										
	Trials	day 1			day 2			day 3				
	Plates	1	2	3	1	2		1	2	3		
	DA	107	30	97	23	25		107	30	97		
	EtOH	21	6	27	2	0		21	6	27		
	N	95	18	69	63	72		95	18	69		
	total	223	54	193	88	97		223	54	193		
	CI	0.39	0.44	0.36	0.24	0.26		0.39	0.44	0.36		
	**Average**	**0.36**										
	**SEM**	**0.03**										
	Conditions	Conditioned										
	Trials	day 1			day 2			day 3				
	Plates	1	2	3	1	2		1	2	3		
	DA	60	25	16	13	4		60	25	16		
	EtOH	31	12	0	0	0		31	12	0		
	N	120	47	59	54	39		120	47	59		
	total	211	84	75	67	43		211	84	75		
	CI	0.14	0.15	0.21	0.19	0.09		0.14	0.15	0.21		
	**Average**	**0.16**									**% LI**	**55**
	**SEM**	**0.01**										

Fig. 5A

wild-type % LI= 99 (from Fig. 3D)
casy-1(tm7 **% LI= 43 (from Fig. 3D)**

glr-1(n2461)

Conditions	Unconditioned										
trials	day 1			day 2			day 3				
Plates	1	2	3	1	2	3	1	2	3		
DA	113	85	114	128	103		77	104			
EtOH	3	3	5	2	3		2	3			
N	15	14	11	10	11		18	18			
total	131	102	130	140	117		97	125			
CI	0.84	0.80	0.84	0.90	0.85		0.77	0.81			
**Average**	**0.83**										
**SEM**	**0.02**										
Conditions	Conditioned										
trials	day 1			day 2			day 3				
Plates	1	2	3	1	2	3	1	2	3		
DA	43	51	79	41	44		35	19			
EtOH	11	2	7	5	16		1	4			
N	34	37	48	44	42		28	17			
total	88	90	134	90	102		64	40			
CI	0.36	0.54	0.54	0.40	0.27		0.53	0.38			
**Average**	**0.43**									**% LI**	**48**
**SEM**	**0.04**										

casy-1(tm718); glr-1(n2461)

Conditions	Unconditioned										
trials	day 1			day 2			day 3				
Plates	1	2	3	1	2	3	1	2	3		
DA	131	64		127	76		80	68			
EtOH	27	29		40	4		5	1			
N	86	69		165	102		5	7			
total	244	162		332	182		90	76			
CI	0.43	0.22		0.26	0.40		0.83	0.88			
**Average**	**0.50**										
**SEM**	**0.12**										
Conditions	Conditioned										
trials	day 1			day 2			day 3				
Plates	1	2	3	1	2	3	1	2	3		
DA	75	44		78	80		49	50			
EtOH	59	28		26	24		8	13			
N	166	80		115	183		26	34			
total	300	152		219	287		83	97			
CI	0.05	0.11		0.24	0.20		0.49	0.38			
**Average**	**0.24**									**% LI**	**51**
**SEM**	**0.07**										

glr-1(n2461); zhEx243.1[glr-1(+)]

Conditions				
trials	day 1			
Plates	1	2		
DA	25	16		
EtOH	0	1		
N	9	11		
total	34	28		
CI	0.74	0.54		
**Average**	**0.64**			
**Standard devia**	**0.39**			
Conditions				
trials	day 1			
Plates	1	2		
DA	5	8		
EtOH	4	2		
N	12	9		
total	21	19		
CI	0.05	0.32		
**Average**	**0.18**		**% LI**	**71**
**Standard devia**	**0.39**		**SEM**	

zhEx243.1[glr-1(+)]

Conditions	Unconditioned								
trials	day 1			day 2			day 3		
Plates	1	2	3	1	2	3	1	2	3
DA	2	9		30			41	60	
EtOH	0	1		0			6	11	
N	6	26		54			4	6	
total	8	36		84			51	77	
CI	0.25	0.22		0.36			0.69	0.64	
**Average**	**0.43**								
**SEM**	**0.10**								

casy-1(tm718); [illegible]

Conditions	Conditioned										
trials	day 1			day 2			day 3				
Plates	1	2	3	1	2	3	1	2	3		
DA	0	4		17	20		23	16			
EtOH	0	2		12	11		17	17			
N	43	47		73	49		21	7			
total	43	53		102	80		61	40			
CI	0.00	0.04		0.05	0.11		0.10	-0.03			
**Average**	**0.05**									**% LI**	**89**
**SEM**	**0.02**										

casy-1(tm718) sibs without *zhEx243.1* array

Conditions	Unconditioned										
Trials	day 1			day 2			day 3				
Plates	1	2	3	1	2	3	1	2	3		
DA	12	19		263			160	208			
EtOH	0	0		23			32	31			
N	24	51		301			20	54			
total	36	70		587			212	293			
CI	0.33	0.27		0.41			0.60	0.60			
**Average**	**0.44**										
**SEM**	**0.07**										
Conditions	Conditioned										
Trials	day 1			day 2			day 3				
Plates	1	2	3	1	2	3	1	2	3		
DA	4	13		33	91		64	136			
EtOH	1	1		6	10		31	99			
N	20	76		258	158		76	162			
total	25	90		297	259		171	397			
CI	0.12	0.13		0.09	0.31		0.19	0.09			
**Average**	**0.16**									**% LI**	**65**
**SEM**	**0.03**										

Fig. 5B

***casy-1(tm718)* Ex[glr-1p::casy-1] line-1**

Conditions	Unconditioned								
Trials	day 1			day 2			day 3		
Plates	1	2	3	1	2	3	1	2	3
DA	4	3		45	26	25	4	8	5
EtOH	0	0		0	0	0	0	0	0
N	2	1		10	6	4	0	6	2
total	6	4		55	32	29	4	14	7
CI	0.67	0.75		0.82	0.81	0.86	1.00	0.57	0.71
**Average**	**0.77**								
**SEM**	**0.06**								

Conditions	Conditioned										
Trials	day 1			day 2			day 3				
Plates	1	2	3	1	2	3	1	2	3		
DA	4	4		12	23	31	5	2	3		
EtOH	0	2		1	1	2	0	3	0		
N	17	14		12	8	28	3	5	7		
total	21	20		25	32	61	8	10	10		
CI	0.19	0.10		0.44	0.69	0.48	0.63	-0.10	0.30		
**Average**	**0.34**									**% LI**	**56**
**SEM**	**0.11**										

***casy-1(tm718)* Ex[glr-1p::casy-1] line-2**

Conditions	Unconditioned								
Trials	day 1			day 2			day 3		
Plates	1	2	3	1	2	3	1	2	3
DA	16	6		10	31	64	22	11	9
EtOH	0	0		0	0	0	9	5	4
N	5	2		1	1	3	6	9	3
total	21	8		11	32	67	37	25	16
CI	0.76	0.75		0.91	0.97	0.96	0.35	0.24	0.31
**Average**	**0.66**								
**SEM**	**0.14**								

Conditions	Conditioned										
Trials	day 1			day 2			day 3				
Plates	1	2	3	1	2	3	1	2	3		
DA	0	0	0	20	12	3	1	1	5		
EtOH	6	3	7	0	2	0	3	2	3		
N	22	47	14	30	19	23	8	17	18		
total	28	50	21	50	33	26	12	20	26		
CI	-0.21	-0.06	-0.33	0.00	0.30	0.12	0.00	-0.05	0.08		
**Average**	**-0.02**									**% LI**	**103**
**SEM**	**0.08**										

p::casy-1] line-3

Conditions	Unconditioned								
Trials	day 1			day 2			day 3		
Plates	1	2	3	1	2	3	1	2	3
DA	9	15	56				8	5	8
EtOH	0	0	0				0	0	0
N	2	2	4				5	2	2
total	11	17	60				13	7	10
CI	0.82	0.88	0.93				0.62	0.71	0.80
**Average**	**0.79**								

***casy-1(tm718)* Ex[glr-1**

**SEM**	**0.05**										
Conditions	Conditioned										
Trials	day 1			day 2			day 3				
Plates	1	2	3	1	2	3	1	2	3		
DA	4	11	20				1	4	1		
EtOH	0	0	1				0	0	0		
N	9	14	23				7	5	4		
total	13	25	44				8	9	5		
CI	0.31	0.44	0.43				0.13	0.44	0.20		
**Average**	**0.32**									**% LI**	**59**
**SEM**	**0.06**										

***casy-1(tm718)* Ex[glr-1p::casy-1] line-4**

Conditions	Unconditioned										
Trials	day 1			day 2			day 3				
Plates	1	2	3	1	2	3	1	2	3		
DA							7	5	2		
EtOH							0	1	0		
N							5	2	0		
total							12	8	2		
CI							0.58	0.50	1.00		
**Average**	**0.69**										
**SEM**	**0.12**										
Conditions	Conditioned										
Trials	day 1			day 2			day 3				
Plates	1	2	3	1	2	3	1	2	3		
DA							0	0	1		
EtOH							3	0	1		
N							15	7	9		
total							18	7	11		
CI							-0.17	0.00	0.00		
**Average**	**-0.06**									**% LI**	**108**
**SEM**	**0.04**										

Average of rescue lines

	Unconditioned					
**Lines**	line 1	line 2	line 3	line 4		
**Average CI**	0.77	0.66	0.79	0.69		
**SEM**	0.06	0.14	0.05	0.12		
	Conditioned					
**Lines**	line 1	line 2	line 3	line 4		
**Average CI**	0.34	-0.02	0.32	-0.05		
**SEM**	0.11	0.08	0.06	0.04	**% LI**	**81**
